# Mineralization Defects in the Primary Dentition Associated With X‐Linked Hypophosphatemic Rickets

**DOI:** 10.1002/jbm4.10463

**Published:** 2021-03-03

**Authors:** Delaney Clayton, Michael B Chavez, Michelle H Tan, Tamara N Kolli, Priscila A Giovani, Kimberly J Hammersmith, Sasigarn A Bowden, Brian L Foster

**Affiliations:** ^1^ Biosciences Division, College of Dentistry The Ohio State University Columbus OH USA; ^2^ Department of Pediatric Dentistry, Piracicaba Dental School University of Campinas Campinas Brazil; ^3^ Division of Pediatric Dentistry, College of Dentistry The Ohio State University Columbus OH USA; ^4^ Department of Dentistry Nationwide Children's Hospital Columbus OH USA; ^5^ Department of Pediatrics, Division of Endocrinology Nationwide Children's Hospital Columbus OH USA; ^6^ College of Medicine The Ohio State University Columbus OH USA

**Keywords:** CEMENTUM, DENTIN, PERIODONTAL TISSUES/PERIODONTIUM, MINERALIZED TISSUE/DEVELOPMENT, HYPOPHOSPHATEMIA, RICKETS

## ABSTRACT

X‐linked hypophosphatemia rickets (XLH) is caused by inactivating mutations in the phosphate‐regulating endopeptidase homolog, X‐linked (*PHEX*) gene, leading to renal phosphate wasting and hypophosphatemia. Dental mineralized tissues are affected by XLH, though tissue‐specific dental mineralization defects have been variably described. We aimed to quantify dental effects in primary teeth of six pediatric patients with XLH (four females and two males, aged 5–12 years). All participants had received conventional treatment of calcitriol and phosphate from initial diagnosis. High‐resolution μCT and histology were employed to analyze 15 exfoliated or extracted primary teeth from patients in comparison with primary control teeth from healthy children. Compared with controls, a third of the teeth from patients with XLH showed reduced enamel density, though enamel thickness was not reduced. Teeth from patients with XLH featured 10% reduction in mean dentin density, though teeth from three affected individuals had dentin densities within normal limits. Dentin mineralization defects were found across all regions of dentin in affected teeth: outer mantle dentin, circumpulpal dentin, and the most recently formed proximal pulpal dentin. Both crown and root dentin thickness appeared reduced in XLH‐affected versus control teeth, with root dentin more severely affected. Interglobular dentin accumulation throughout the circumpulpal dentin was evident by both μCT and histology (585‐fold volume increase and 45‐fold area increase, respectively). Histology also revealed substantially wider predentin and decreased acellular cementum thickness in XLH‐affected versus control teeth, though thinner cementum appeared to include functionally oriented Sharpey's fibers except in the most severely affected individuals. These data provide quantitative analyses of dental mineralization defects associated with XLH, finding a wide range of severity, with dentin more significantly and consistently defective across the spectrum of XLH.

## Introduction

X‐linked hypophosphatemic rickets (XLH; OMIM# 307800) caused by inactivating mutations in *PHEX* (phosphate‐regulating endopeptidase homolog, X‐linked), is the most common form of inherited rickets at approximately 1:20,000 births.^(^ ^1^, ^2^ ^)^ XLH is characterized by renal phosphate wasting caused by increased fibroblast growth factor 23 (FGF23), leading to inappropriately low 1,25‐dihydroxyvitamin D and hypophosphatemia, contributing to mineralization disturbances. Skeletal manifestations of XLH include rickets and osteomalacia, which result in lower extremity deformity (genu varum or genu valgum), short stature, pain, enthesopathy, and craniofacial effects.^(^ ^3^ ^)^ XLH causes dental defects that lead to spontaneous tooth abscesses, pulpal necrosis, and increased prevalence of periodontal disorders that are not well‐defined.^(^ ^3^, ^4^, ^5^, ^6^, ^7^, ^8^, ^9^, ^10^ ^)^

The dentoalveolar complex features four unique mineralized tissues: enamel, dentin, cementum, and alveolar bone. Although odontogenesis differs from osteogenesis in several respects, mineralization of enamel, dentin, and cementum occurs through mechanisms that parallel skeletal mineralization and are susceptible to developmental disturbances of mineral metabolism.^(^ ^4^, ^9^, ^11^ ^)^ Because of the variable descriptive approaches used in previous reports, some aspects of XLH‐associated dental pathology remain unclear, particularly how dental effects correspond to genetic alterations and severity of biochemical and skeletal alterations, and how each dental mineralized tissue is comparatively affected. New approaches for more sophisticated quantitative analysis may provide additional insights to previous reports focusing on tooth morphology and histology.^(^ ^8^, ^10^, ^12^, ^13^ ^)^ With an effective FGF23‐targeting antibody therapy (burosumab or Crysvita; Ultragenyx Pharmaceutical) approved for treatment of children and adults with XLH,^(^ ^14^, ^15^, ^16^ ^)^ it is important to understand the nature of dental defects to evaluate efficacy of treatment. We hypothesized that enamel, dentin, and cementum in primary teeth of affected individuals would reflect XLH‐associated mineralization defects compared with healthy controls.

## Subjects and Methods

### Study participants

Written informed consent was obtained from legal guardians, and assent was obtained from six pediatric patients with XLH enrolled under an institutional review board protocol approved by The Ohio State University and Nationwide Children's Hospital (Columbus, OH). Enrolled patients donated exfoliated or extracted primary teeth (*n* = 15), and their medical and dental records were reviewed. Nine primary teeth from five approximately age‐matched healthy control individuals (aged ~6‐11 years) were analyzed for comparison.

### Micro‐computed tomography

μCT was used to analyze teeth. Fixed samples were scanned in a μCT 50 (Scanco Medical) at 70 kVp, 76 μA, 0.5‐mm Al filter, with 900‐ms integration and 10‐μm voxel dimension. Reconstructed images were analyzed using AnalyzePro 1.0 (AnalyzeDirect) as described previously.^(^ ^17^ ^)^ Images were oriented anatomically using the midsagittal slice, where the mesial and distal cementum‐enamel junctions (CEJs) were used to identify an axis perpendicular to the root. Oriented scans were calibrated to a standard curve of five known hydroxyapatite (HA) densities (mg/cm^3^ HA). Dentin was segmented at 650–1600 mg/cm^3^ HA, and enamel was segmented above 1600 mg/cm^3^ HA. Acellular cementum was segmented as previously described.^(^ ^17^, ^18^ ^)^ Briefly, a median filter with a kernel size of 11 was applied. Acellular cementum was then segmented between 450–1050 mg/cm^3^ HA with manual corrections to exclude softer dentin that was highlighted adjacent to the pulp. The segmentation map was then loaded back onto the original calibrated image and used as a mask to highlight any cementum under this mask with a density over 650 mg/cm^3^ HA. Quantitative analysis was then performed on the original calibrated image.

Primary teeth are challenging to analyze because of different degrees of enamel attrition and root resorption; therefore, regions of interest (ROIs) for thickness measurements were selected within the cervical part of the tooth near the cementum–enamel junction (CEJ). For crown dentin and enamel thickness and interglobular dentin area, we defined the ROI by identifying the CEJ in the axial plane as the first slice beginning when enamel made a complete ring, and then moved coronally 50 slices (0.5 mm). Interglobular dentin was defined as the volume within the crown dentin ROI that was <650 mg/cm^3^ HA. For root dentin thickness, we identified the most apical extension of enamel in the axial orientation and moved apically 150 μm to outline an ROI consisting of 0.5 mm. Enamel and dentin thicknesses were measured using a cortical bone thickness algorithm for the defined ROIs that was adapted from Bouxsein and colleagues, where average thickness of the cylinder over the z‐stack is calculated.^(^ ^19^ ^)^ Subdivision of dentin mineral density was performed in the root dentin ROI by designating mantle dentin (outermost 150 μm, excluding cementum), proximal pulpal dentin (innermost 150 μm), and circumpulpal dentin (all dentin between mantle and proximal pulpal regions) as described previously.^(^ ^18^ ^)^ There is not a consensus on mantle dentin thickness in humans. Some texts and publications have reported a layer on the scale of tens of μm, whereas Goldberg and colleagues reported a less mineralized outer dentin layer of approximately 200 μm.^(^ ^20^ ^)^ Proximal pulpal dentin is not a previously recognized anatomical form of dentin but is rather a term we used to interrogate the most recently formed mineralized dentin. Acellular cementum thickness was determined using cortical bone thickness algorithms from the most apical 25–50 slices that formed a hollow cylinder of acellular cementum (depending on whether sufficient root structure remained).

### Histology

Teeth were fixed in 10% neutral buffered formalin, demineralized in a formic acid and formaldehyde solution (Polysciences, Inc), paraffin embedded, and sectioned at 6 μm. Tissue sections were stained with hematoxylin and eosin, toluidine blue, or picrosirius red as previously described.^(^ ^17^, ^21^ ^)^ Histomorphometry was performed on stained histological sections using imageJ software (version 1.46r; National Institutes of Health, Bethesda, MD) to measure predentin thickness (×20 magnification) and cementum thickness (×40 magnification). Interglobular dentin defects were assessed in imageJ by converting ×20 color images of dentin (*n* = 3 sections per tooth) to 16‐bit grayscale, adjusting the threshold of the histograms, converting to binary (black/white) mode, and measuring percentage of black area (equivalent to normal, mineralized dentin) over the total area in the image.

### Statistical analysis

The mean ± SD from XLH and control teeth measurements is shown in graphs with 95% CIs generated from control teeth. Graphs were made using Prism version 7.04 (GraphPad Software).

## Results

### Medical histories of enrolled patients with XLH

Table 1 summarizes the characteristics of the study population (labeled as patients 1–6). Participants included four females and two males aged 5–12 years at the time of clinical data and history collection. Diagnosis of XLH occurred at ages 9 months to 5 years. *PHEX* mutations were identified in five of six individuals, with all genetic alterations being novel. These included an intronic variant (patient 2: C.850‐2 A > G), a missense mutation (patient 3: c.1544A > C; p.Q515P), splicing mutation (patients 4 and 5, who are siblings: IVS2‐c.187 + 1delG), and a nonsense mutation resulting in a premature stop codon (patient 6: c.1368G > A p.W456X). Patient 1 was diagnosed by clinical presentation, strong family history, and biochemical and radiographic findings consistent with XLH; therefore, no genetic testing was performed. All participants exhibited hypophosphatemia with clinical and radiographic signs of rickets. All but one had elevated alkaline phosphatase (ALP) levels, a marker for active rickets, whereas some also had elevated PTH and FGF23 levels. Most patients presented with genu varum (bowed legs); two patients (patients 1 and 4) presented with genu valgum (knock‐knee deformity). Height z‐scores for all patients were >1 SD below average, ranging from −1.22 to −3.78. Only patient 1 required a surgical intervention with guided growth surgery to correct her leg deformity. Nephrocalcinosis, a treatment complication from calcitriol and phosphate, was found in patients 2 and 6.

**Table 1:** Demographic, Genetic, and Biochemical Results, Treatment Regimens Including Surgical Intervention to Correct Leg Deformity, Treatment Complication (Nephrocalcinosis), and Teeth Analyzed for Enrolled Individuals

Parameter	Patient 1	Patient 2	Patient 3	Patient 4	Patient 5	Patient 6
Sex, M/F	F	M	F	F	M	F
Age at time of data collection, y	10	5	9	12	6	12
Age at diagnosis, y	5	0.9	3.3	0.9	1.3	2.8
Height Z‐score	−1.6	−1.8	−1.2	−3.4	−2.5	−1.9
Genetic mutation(s) in *PHEX*	N/A	C.850–2 A > G IVS7‐2 A > G	c.1544A > C p.Q515P	IVS2‐ c.187 + 1delG	Sibling of patient 4 (presumed same mutation)	c.1368G > A p.W456X
Serum calcium (normal 8–10.5 mg/dL)	10.1	9.6	9.6	10.2	9.6	9.0
Serum phosphorus (age‐dependent reference range; mg/dL)	At diagnosis: **3.4** (4.2–6.5) Age 9 y^a^: **2.5** (3.7–5.6)	Age 3 y^a^: **3.1** (4.5–5.5)	At diagnosis: **3.3** (4.2–6.5) Age 8 y^a^: **3.4** (3.7–5.6)	At diagnosis: **3.3** (4.2–6.5) Age 11 y^a^: **2.3** (3.7–5.6)	At diagnosis: **2.8** (4.2–6.5) Age 5y 9 mo^a^: **2.8** (4.1–5.9)	At diagnosis: **3.0** (3.7–5.6) Age 11 y^a^: **2.7** (3.7–5.6)
Serum alkaline phosphatase (age‐ and sex‐ dependent reference range; U/L)	At diagnosis: **610** (65–500) Age 9 y^a^: **449** (151–342)	Age 3 y^a^: **322** (100–320)	At diagnosis: **667** (85–360) Age 8 y^a^: **480** (151–342)	At diagnosis: **481** (55–380) Age 11 y^a^: 345 (137–424)	At diagnosis: 356 (85–360) Age 5y 5 mo^a^: 295 (151–342)	At diagnosis: **506** (85–360) Age 11 y^a^: 336 (137–424)
Serum 25‐hydroxy vitamin D at diagnosis (normal 20–40 ng/mL)	44	N/A	27	55	37	55
Serum 1,25 dihydroxy vitamin D at diagnosis (normal 15–90 pg/mL)	34	N/A	51	58	38	68
PTH (normal 10–65 pg/L)	At diagnosis: 54 Age 9 y: 51	Age 3 y^a^: 44.4 (18–80)	At diagnosis: 51 Age 8 y^a^: 23	At diagnosis: 56 Age 11 y^a^: 53	At diagnosis: **80** Age 5 y 5 mo^a^: 21	At diagnosis: **88** Age 11 y^a^: 64
Serum FGF23 (normal ≤230 RU/mL)	169	N/A	158	N/A	167	**264**
Treatments with age at the start of treatment	5 y: Calcitriol and phosphate 9 y 5 mo: Burosumab	0.9 y: Calcitriol and phosphate 4 y: Burosumab	3 y: Calcitriol and phosphate 8 y: Burosumab	0.9 y: Calcitriol and phosphate 11 y 2 mo: Burosumab	1.3 y: Calcitriol and phosphate 5 y 9 mo: Burosumab	2 y 8 mo: Calcitriol and phosphate 11 y: Burosumab
Guided growth surgery of legs	Yes	No	No	No	No	No
Nephrocalcinosis	No	Yes	No	No	No	Yes
Teeth analyzed, No.	1	3	3	1	6	1
Teeth types, A‐T	D	O, P, Q	F, N, O	P	C, D, F, G, N, P	C

*Note*: The ages in years denote the time point of the majority of lab testing performed for respective patients. Bold font indicates values outside of the normal range.

Abbreviations: FGF23, fibroblast growth factor 23; *PHEX*, phosphate‐regulating endopeptidase homolog, X‐linked.

^a^ Indicates laboratory results obtained while patients were on calcitriol and phosphate treatment.

Conventional treatment of oral calcitriol and phosphate was initiated for all patients shortly after their diagnoses. All of them received conventional treatment over a range of 4–10 years before changing to the new anti‐FGF23 antibody therapy with burosumab (Crysvita) within the past year or less at the time of data collection. Primary teeth analyzed in this report were exfoliated or extracted while participants were on conventional therapy; therefore, burosumab would have no bearing on results.

### Dental histories of enrolled patients with XLH

**Fig 1:**
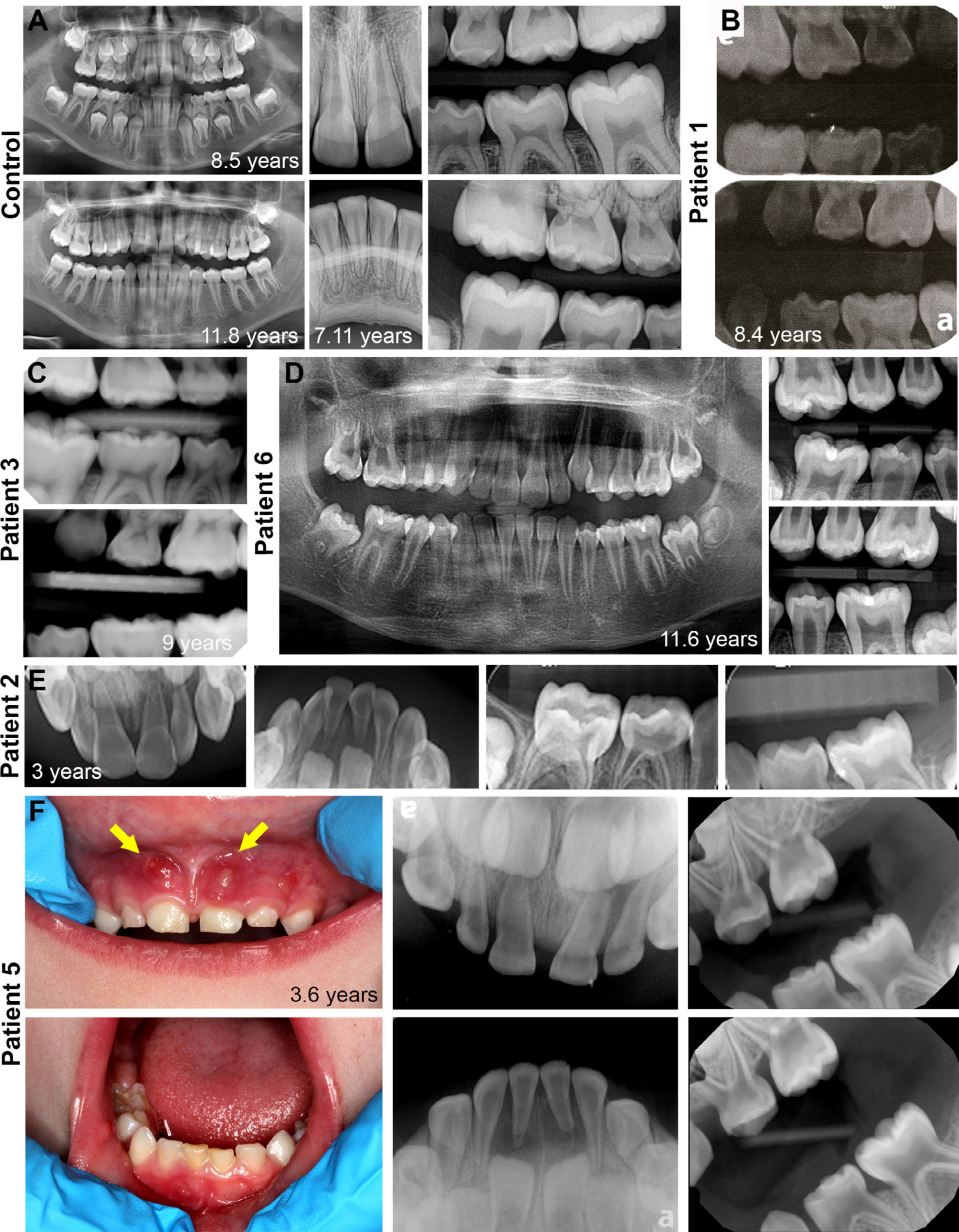
Clinical and radiographic effects of X‐linked hypophosphatemia rickets (XLH) on the dentition. (*A*) Dental panoramic, periapical, and bitewing radiographs from a representative healthy control individual at ages of 7 years 11 months, eight years five months, and 11 years 8 months. (*B*) Bitewing radiographs from XLH patient 1 aged 8 years 4 months showing normal enamel, dentin, and pulp chamber appearance. (*C*) Bitewing radiographs from patient 3 at 9 years of age showing normal enamel, dentin, and pulp chamber appearance. (*D*) Panoramic and bitewing radiographs from patient 6 aged 11 years 6 months shows relatively normal pulp chambers and canals in the adult dentition. (*E*) Periapical and bitewing radiographs from patient 2 aged 3 years indicate thin enamel, thin dentin, and enlarged pulp chambers in anterior teeth. (*F*) Oral photographs from patient 5 aged 3 years 6 months show the presence of fistulas (yellow arrows) near maxillary incisors. Periapical and bitewing radiographs show thin enamel, thin dentin, and enlarged pulp chambers.

Dental records were reviewed, excluding patient 4 whose records were unobtainable. Patients 1 and 3 had unremarkable dental histories in terms of tooth eruption and exfoliation, overall oral health, and treatments, whereas patients 2, 5, and 6 had notable dental records. Compared to normal development of primary dentition (Fig. 1*A*), patients 1 and 3 had no overt signs of dental defects typically associated with XLH (e.g., thin dentin, abnormal pulp chambers, extensive caries, and abscesses; Fig. 1*B*,*C*).

The dental history for patient 6 was significant for extensive caries. At age 3 years, she was referred to a pediatric dentist for operative dentistry under general anesthesia and had eight carious teeth treated. Patient 6 provided no radiographic images other than a panoramic and bitewing radiographs from age 11 years 6 months, showing widened pulp chambers and canals in the early adult dentition (Fig. 1*D*).

Patients 2 and 5, the two males in the study population, had the most remarkable dental histories. Patient 2 had wide pulp chambers in the anterior teeth with more normal appearing molars (Fig. 1*E*). Patient 5 had extremely wide pulp chambers and thin dentin in maxillary and mandibular molars and incisors (Fig. 1*F*). Both individuals reported extractions or premature loss of teeth; patient 2 lost 5 teeth and patient 5 lost 10 teeth prior to expected exfoliation times.

The dental history for patient 2 was significant for fistulas and draining abscesses in maxillary and mandibular incisor regions. The etiology of the abscesses was speculated to be large pulp chambers with thin dentin that increased the likelihood of pulp exposure and necrosis. The following month, teeth O and P were lost caused by minor trauma during play. The teeth were abscessed and mobile prior to injury, likely facilitating avulsion. After loss of O and P, the abscesses on the mandibular anterior region subsided. The abscesses above teeth E and F persisted and after several months those teeth were extracted. One other tooth (Q) was avulsed during play, though its mobility or infection status was unknown. Patient 2 spontaneously lost teeth at 3 years 9 months and 4 years 5 months of age, whereas the normal range for incisor exfoliation is 6‐–7 years old.

Patient 5 underwent extensive full‐mouth rehabilitation at age 3.5 years 5 months to address enamel attrition and abscesses associated with rickets (Fig. 1*F*). The eight primary incisors were extracted, and eight primary molars received pulpotomies and stainless‐steel crowns. The four primary canines received strip crowns with no tooth preparation. At age 4 years 11 months, he was admitted to the hospital for dental infection and facial swelling. The week prior, he had tooth B extracted and was prescribed amoxicillin but pain and swelling persisted. The extraoral exam revealed erythematous swelling to the right of the canine space extending to the right periorbital region. The intraoral exam revealed vestibular obliteration from the distal aspect of tooth B to the mesial of tooth C, with tooth C positive to percussion and palpation tests. Tooth C was extracted at the parents' request and tooth S was extracted because of internal root resorption and furcal radiolucency.

### Enamel and dentin defects associated with XLH in more severely affected individuals

**Fig 2:**
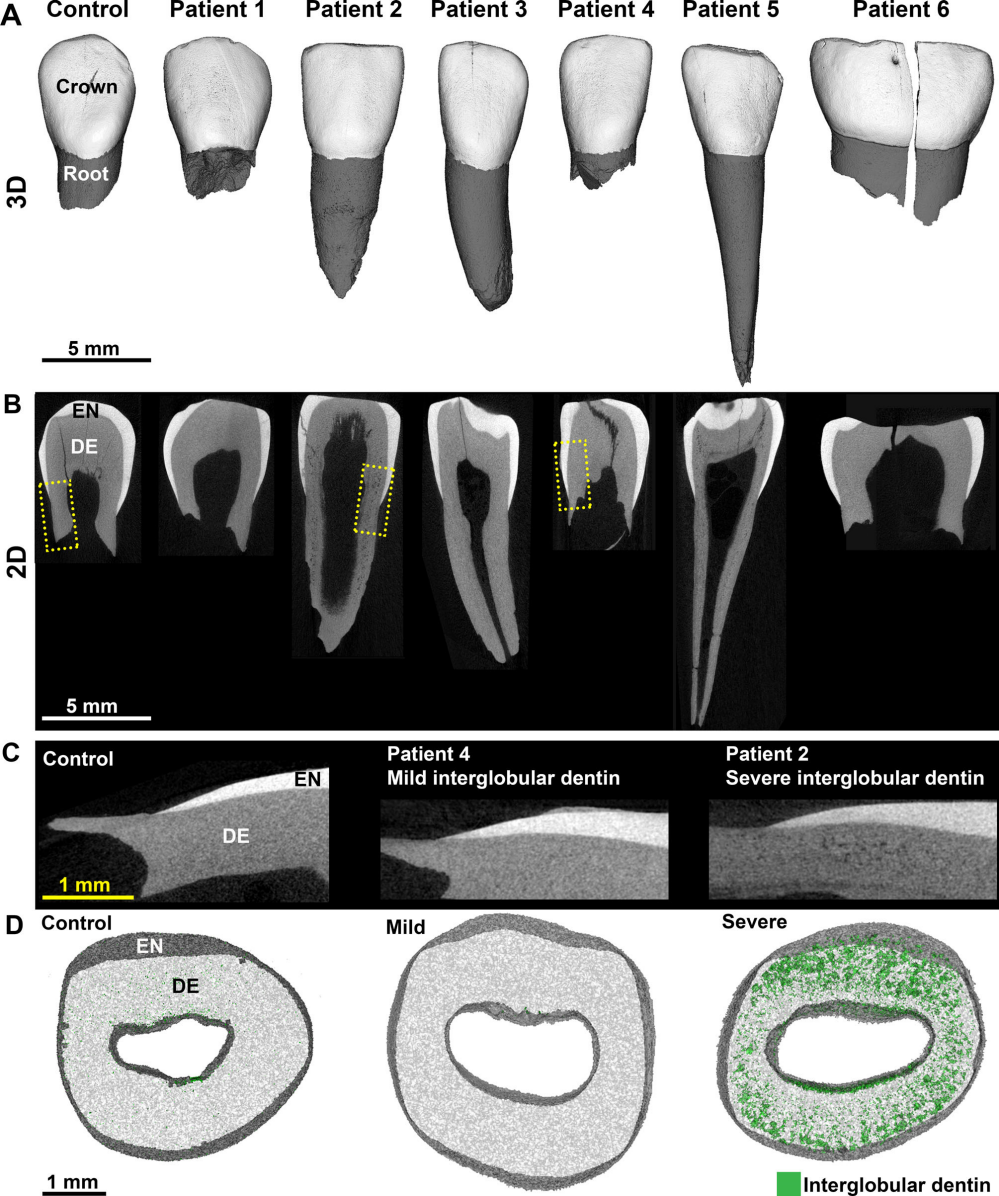
Primary teeth from patients with X‐linked hypophosphatemia rickets (XLH). Representative (*A*) three‐dimensional and (*B*) two‐dimensional (2D) renderings from μCT scans showing representative primary teeth from a healthy control individual and patients 1–6. Enamel (EN) is shown in white and dentin (DE) is shown in gray. Yellow dotted boxes in panel (B) indicate areas shown at higher magnification in (*C*) illustrating mild and severe levels of hypomineralized interglobular dentin (radiolucent regions). (*D*) Transverse 2D images of representative teeth showing hypomineralized interglobular dentin (<450 mg/cm^3^) where DE is shown in white to visualize interglobular dentin in green. Mildly affected patients with XLH exhibited little to no obvious interglobular dentin. Severely affected individuals had substantial accumulation of interglobular dentin.

A total of 15 teeth from the six patients with XLH (Table 1) were analyzed by high‐resolution μCT (Fig. 2*A*,*B*). Some patients lost primary teeth according to the expected schedule of exfoliation; however, dental records (as outlined above) indicated that some teeth were extracted because of abscesses, and others were reported to have been lost prematurely. Two‐dimensional (2D) images are notable for evidence of interglobular dentin—hypomineralized regions of dentin where HA calcospherites did not merge into a united mineralization front (Fig. 2*C*). Healthy control teeth exhibited little to no detectable interglobular dentin, whereas among XLH teeth some exhibited mild levels of interglobular dentin (not different from controls) and others displayed severe accumulation of hypomineralized regions (Fig. 2*C; interglobular* dentin indicated by green shading in Fig. 2 *D*).

**Fig 3:**
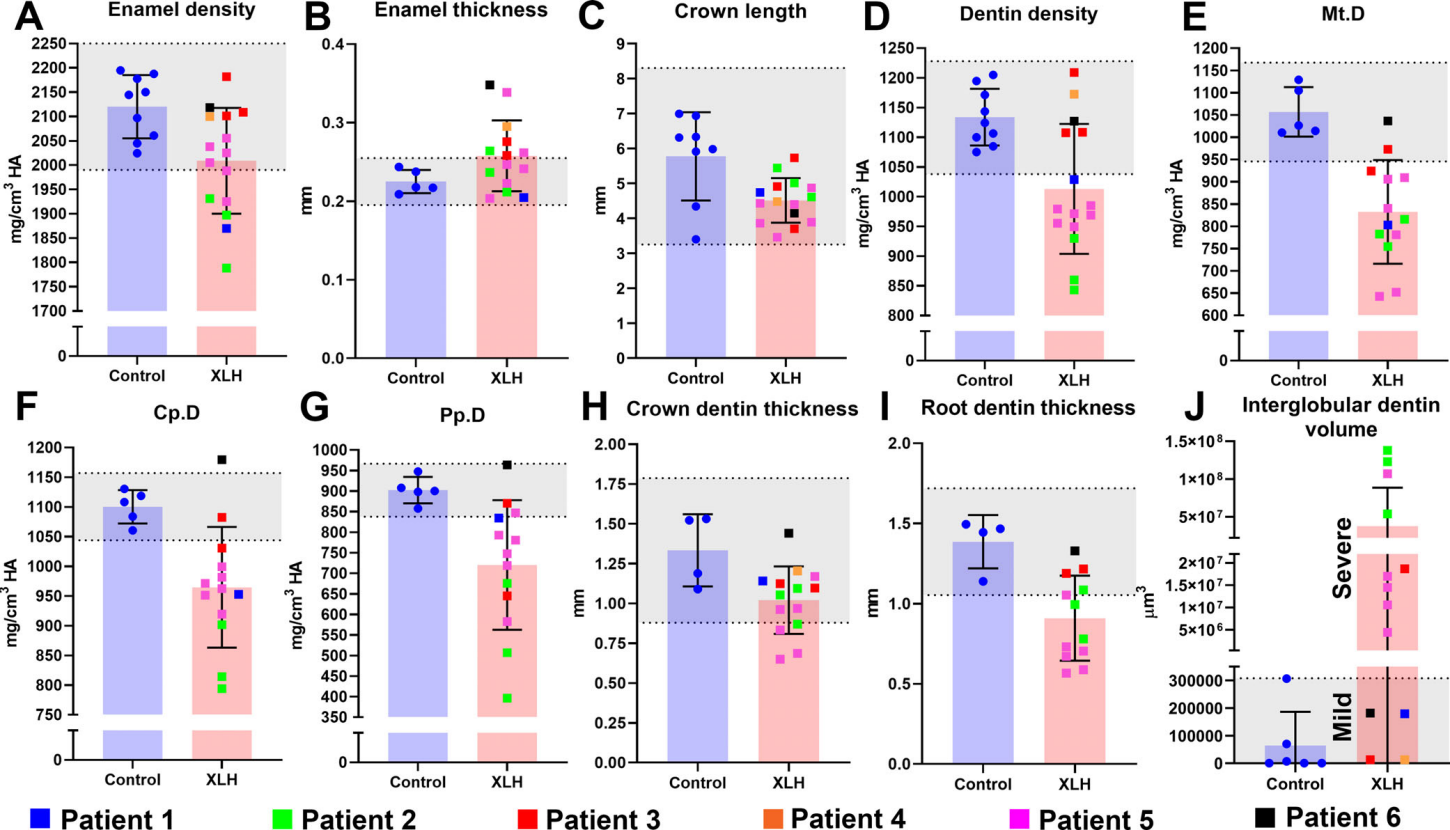
Mineralization defects associated with X‐linked hypophosphatemia rickets (XLH) in primary teeth. (*A*‐*I*) Individual values, means, and SDs from μCT analyses of dental tissues of patients with XLH versus control individuals. Control 95% CIs are shaded in gray. Patients 1–6 are color coded to recognize patterns in data across measurements. Mt.D = Mantle dentin (outermost); Cp.D = circumpulpal dentin (middle); Pp.D = proximal pulpal dentin (inner and last formed). (*J*) Measured volumes of interglobular dentin. Values for mildly affected patients with XLH fall within the 95% CI, and values for severely affected patients are substantially elevated above the 95% CI.

Quantitative analysis determined densities and thicknesses of enamel and dentin. Mean XLH enamel density fell within the 95% CI range of controls; however, enamel density for patients 1, 2, and 5 was below the 95% CI, indicating substantial enamel mineralization defects (Fig. 3*A*). All patients fell within the 95% CI, indicating normal enamel thickness (Fig. 3*B*). Mean crown length in XLH‐affected teeth was reduced, though the effect was mild (Fig. 3*C*).

XLH‐affected teeth exhibited substantial dentin defects with 10% reduction in mean dentin density that fell below the control 95% CI (Fig. 3*D*). Teeth from patients 1, 2, and 5 placed entirely below the 95% CI. We aimed to determine localization of dentin defects associated with XLH and found that mantle dentin, circumpulpal dentin, and proximal pulpal (essentially outermost, middle, and innermost, respectively) all showed dramatic decreases in average mineral density in XLH teeth (Fig. 3*E*‐*G*). Both crown and root dentin thicknesses were reduced compared with controls, with roots more severely affected (Fig. 3*H*,*I*). Patients 2 and 5 were the most severely affected. Three‐dimensional quantification of interglobular dentin volume confirmed an average 585‐fold mean increase with mild (no different from controls; patients 1, 3, 4, and 6) or severe effects (patients 2 and 5, and one tooth from patient 3) noted (Fig. 3*J*).

### Interglobular dentin accumulation and cementum defects associated with XLH

**Fig 4:**
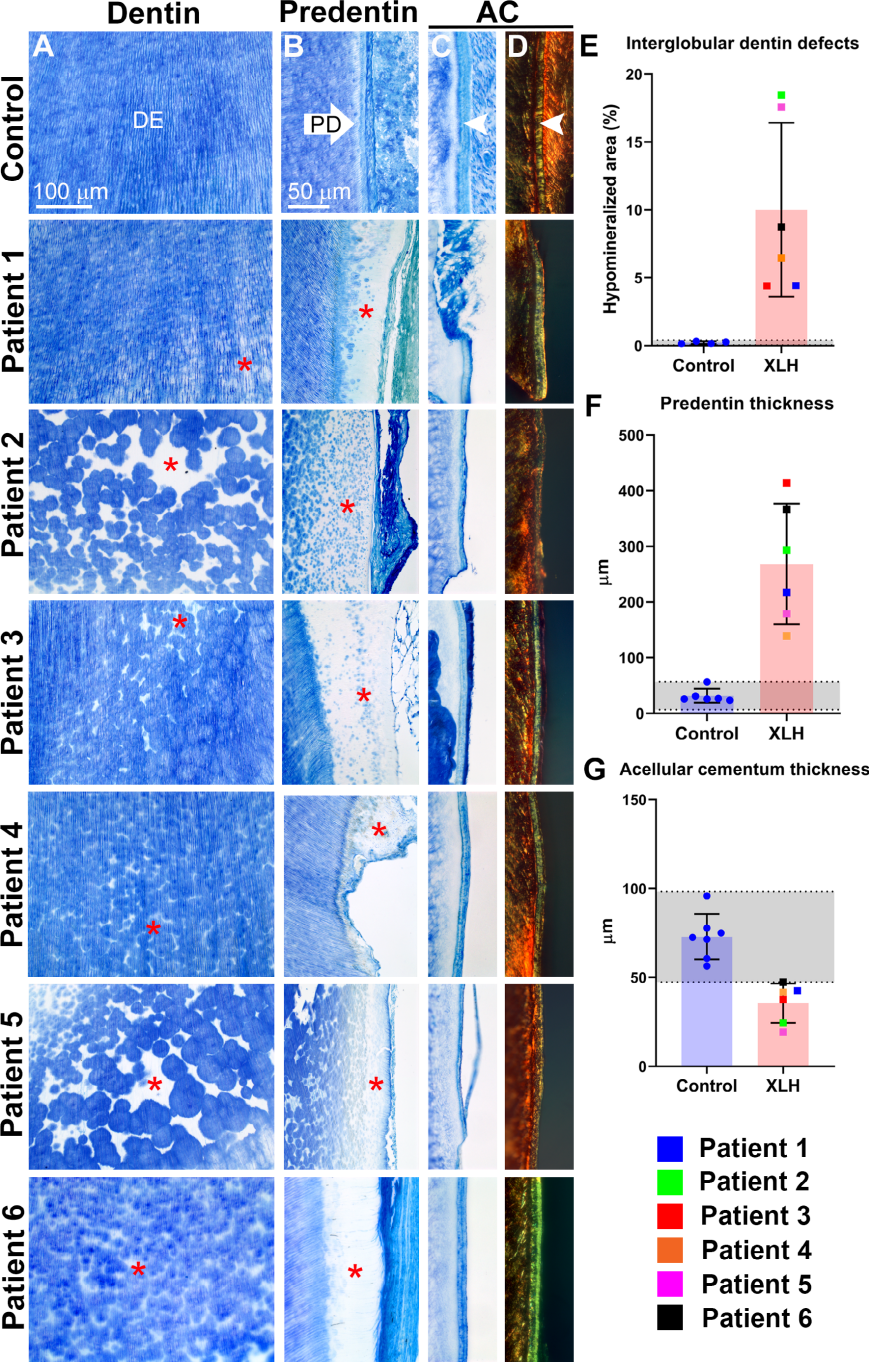
Histological features of dentin and cementum in primary teeth associated with X‐linked hypophosphatemia rickets (XLH). (*A*) Toluidine blue staining revealed interglobular dentin defects (white spaces, indicated by red stars) in all teeth from all patients with XLH with varying severity. (*B*) Predentin in XLH‐affected teeth is wide with an erratic border compared with controls (red stars). (*C*) Acellular cementum (AC; white arrowhead in control section) was observed on the root surfaces of all XLH‐affected teeth, though thinner for patients 2 and 5. (*D*) Picrosirius red staining observed under polarized light microcopy showed organized Sharpey's fibers within the cementum layer. Teeth from patients 2 and 5 exhibited reduced signal and organization. (*E*) Quantitative analysis confirmed in XLH‐affected teeth 45‐fold increased interglobular dentin and eightfold increased predentin thickness based on toluidine blue staining patterns, whereas mean cementum thickness was decreased 50% in XLH teeth, and individual measurements fell below the 95% CI established by healthy controls.

Histology was performed to further examine dentin and cementum organization. Toluidine blue staining revealed interglobular dentin defects in all teeth from all patients with XLH, though severity varied widely between individuals (Fig 4*A*). Compared with controls, where predentin was predictably narrow, XLH‐affected teeth exhibited a large predentin region with an erratic border, often with interglobular patterns adjacent to or merging with the predentin zone (Fig. 4*B*). Cementum was observed on the root surfaces of all XLH‐affected teeth; however, teeth from patients 2 and 5 revealed a much thinner layer (Fig. 4*C*). Picrosirius red staining observed under polarized light microcopy confirmed embedded Sharpey's fibers within the cementum layer (Fig. 4*D*). Notably, patients 2 and 5 exhibited more poorly developed collagen fiber organization within cementum. Quantitative analysis confirmed in XLH teeth a 45‐fold increased interglobular dentin area and an eightfold increased predentin thickness, while mean cementum thickness was decreased 50% in XLH‐affected teeth, and all individual measurements fell below the 95% CI established by healthy controls.

## Discussion

We report high‐resolution quantitative μCT analysis of primary teeth from six patients with XLH, in parallel with genetic, biochemical, and skeletal effects. We found a range of XLH dental manifestations, with some patients reporting unremarkable dental histories and few quantifiable effects on primary teeth, and others exhibiting substantially affected oral health and severe defects in dental tissues. Although enamel density was reduced in more severely affected individuals, dentin was more significantly and consistently defective across the spectrum of XLH. We found mineralization defects across all regions of dentin including reduced mineral density, increased accumulation of interglobular dentin, and increased predentin width. Though cementum thickness was consistently reduced in XLH, dental history and selective staining suggested functional periodontal attachment in most patients, though one severely affected child prematurely lost multiple teeth where defective periodontal attachment may have contributed.

### Quantifying the dental manifestations of XLH

Numerous case reports and reviews spanning several decades have summarized dental defects associated with XLH.^(^ ^3^, ^4^, ^5^, ^6^, ^7^, ^8^, ^9^, ^10^, ^12^, ^13^, ^22^, ^23^, ^24^, ^25^, ^26^, ^27^, ^28^, ^29^, ^30^, ^31^ ^)^ Descriptions of XLH‐associated dental pathology consistently highlight dentin defects, particularly accumulation of interglobular dentin. Increased interglobular dentin indicates the presence of unmerged calcospherites, discrete mineralization foci that normally would grow and merge into a unified mineralization front. Dentin hypomineralization is considered responsible for most of the clinical manifestations described, including spontaneous dental abscesses and pulpal necrosis. However, enamel defects or microcracks, thin dentin, wide predentin, enlarged pulp chambers, prominent pulp horns, altered root size and shape, alveolar bone hypomineralization, cementum abnormalities, and increased prevalence of periodontal disorders are also described. Inconsistencies in prior reports may arise from reliance on subjective qualitative and descriptive methods, different criteria or level of detail in evaluations, and/or inherent variability in the disease and its effects. We aimed to develop a quantitative approach to analyze primary teeth from individuals affected by XLH, in part to better define XLH‐associated dental defects.

A surprisingly wide range of dental defect severity was discovered in the enrolled patients. Some had unremarkable dental histories (patients 1 and 3); others experienced substantial disruptions in oral health such as abscesses and pulpal necrosis and underwent extensive and invasive procedures, including extractions and placement of stainless‐steel crowns (patients 2, 5, and 6). For the most part, these significant dental histories coincided with defects documented by μCT and histology. Patients 3, 4, and 6 registered in the normal range for almost all metrics, indicating that dental mineralization was largely undisturbed. This is curious for patient 6 who experienced severe caries but exhibited no quantitative defects in primary tooth enamel or dentin. In contrast, patients 2 and 5 exhibited profound defects, whereas patient 1 had variable effects depending on parameter measured and tooth. Patients 2 and 5 are males carrying hemizygous *PHEX* mutations that predispose to more severe disease, explaining more dramatic dental defects. Patient 1 is a female diagnosed and beginning conventional treatment at a substantially later age than the other patients. She exhibited some mineralization defects detectable by μCT and histology, but no clinically significant adverse oral health effects.

XLH broadly impacted all the dental mineralized tissues in primary teeth. Enamel density appeared reduced in teeth from patients 1, 2, and 5. XLH features hypophosphatemia and reduced 1,25‐dihydroxyvitamin D levels, and both of these changes can directly affect ameloblast functions as shown by nutritional and genetic mouse studies.^(^ ^3^, ^32^, ^33^, ^34^ ^)^ Ameloblasts have been reported to have low levels of PHEX protein localization,^(^ ^35^ ^)^ though another study found no detectable *Phex* mRNA,^(^ ^36^ ^)^ making the exact mechanism for enamel defects in XLH unclear. The *Hyp* mutant mouse model of XLH carries inactivating *Phex* mutations and phenocopies biochemical and skeletal features of XLH.^(^ ^1^ ^)^ Dental defects in *Hyp* mice include enamel hypoplasia with no detectable difference in enamel density versus controls.^(^ ^36^ ^)^ These differences in human versus mouse enamel defects warrant further investigation.

Dentin was the most severely affected hard tissue, consistent with numerous prior reports.^(^ ^6^, ^8^, ^9^, ^10^, ^12^, ^13^, ^22^, ^24^, ^25^, ^26^, ^27^, ^28^, ^29^, ^30^, ^31^ ^)^ Several patients exhibited reduced dentin density and thickness, and hypomineralization was detected across mantle, circumpulpal, and proximal pulpal dentin regions. This subdivision is of interest because of developmental and mechanistic implications. Mantle dentin is reported to depend on matrix vesicle initiated mineralization, whereas mineralization of circumpulpal dentin is guided by phosphoproteins such as dentin sialoprotein and dentin phosphoprotein.^(^ ^20^, ^37^, ^38^, ^39^ ^)^ All dentin regions appear to be adversely and similarly affected, indicating a universal necessity for PHEX function in all stages of dentinogenesis. Three‐dimensional quantification of interglobular dentin indicated defects in some patients but normal levels in others, whereas 2D measurements indicated all patients harbored hypomineralized areas. Differences in μCT versus histological measurement of interglobular dentin may result in part from resolution differences. μCT was performed with a 10‐μm voxel size, so would be limited to identifying larger hypomineralized regions measuring at least several voxels. Light microscopy at ×20 magnification has the benefit of resolution below 1 μm; therefore, it detects substantially smaller hypomineralized regions than μCT. All patients also showed evidence of wide and erratic predentin borders, supporting ongoing dentin mineralization defects even under conventional treatment.

Few reports on XLH have analyzed cementum. Reduced thickness of acellular cementum was reported in a study on the permanent dentition in XLH,^(5^ ^)^ and cellular cementum mineralization was described as defective.^(^ ^40^ ^)^ We found consistently reduced acellular cementum in all XLH‐affected teeth. Picrosirius red staining showed functionally oriented Sharpey's fibers that suggest periodontal attachment would be maintained. However, more severely affected patients exhibited the thinnest cementum in combination with poorly organized collagen fibers within cementum, mirroring more severe effects on enamel and dentin. Premature exfoliation of teeth is generally not described for XLH, though extractions for necrotic teeth are common. Patient 2 lost teeth prematurely, likely because of the chronic abscesses causing bony destruction and subsequent tooth mobility. Increased mobility facilitates avulsions with only minor forces of trauma. This sort of premature and spontaneous loss of fully rooted teeth is unusual for XLH but very familiar for hypophosphatasia, another inherited mineralization disorder where lack of acellular cementum results in loss of periodontal attachment. The *Hyp* mouse model of XLH has thin acellular cementum, regions of periodontal ligament detachment, and significantly disturbed periodontal mechanical properties.^(^ ^36^ ^)^ These types of accumulated periodontal defects may increase the risk of periodontal disease later in life in individuals with XLH.^(^ ^5^ ^)^

## Conclusions

In summary, we report an approach to quantify dental mineralization defects associated with XLH using high‐resolution μCT. Limitations include the small study size and variable ages of patients, though these are perpetual challenges in the study of rare genetic disorders. Because of the availability of exfoliated/extracted XLH teeth, analysis focused mostly on incisors, so conclusions should be generalized with caution to the entire dentition. These quantitative data describing dental mineralization defects associated with XLH may contribute to a more in‐depth understanding of pathological mechanisms, and the approaches used may be of use for other skeletal and dental disorders.

## Conflict of Interest

The authors declare no potential conflicts of interest with respect to the authorship and/or publication of this article.

## Author Contributions

**Delaney Clayton:** Formal analysis; investigation; visualization; writing‐original draft; writing‐review & editing. **Michael Chavez:** Conceptualization; formal analysis; investigation; methodology; visualization; writing‐review & editing. **Michelle Tan:** Formal analysis; visualization; writing‐review & editing. **Tamara Kolli:** Formal analysis; visualization; writing‐review & editing. **Priscila Giovani:** Formal analysis; visualization; writing‐review & editing. **Kimberly Hammersmith:** Methodology; visualization; writing‐review & editing. **Sasigarn Bowden:** Conceptualization; methodology; supervision; writing‐review & editing. **Brian Foster:** Conceptualization; formal analysis; funding acquisition; investigation; methodology; supervision; visualization; writing‐original draft; writing‐review & editing.

### PEER REVIEW

The peer review history for this article is available at https://publons.com/publon/10.1002/jbm4.10463.
